# Challenges and Advances in the Simulation of Targeted Covalent Inhibitors Using Quantum Computing

**DOI:** 10.1021/acs.jpclett.5c01680

**Published:** 2025-08-12

**Authors:** Shayantan Chaudhuri, Bang C. Huynh, Ross Amory, David M. Rogers, Aiden Cranney, Luis A. Martínez-Martínez, Tommaso Macrì, Glenn Jones, Evan Sheridan, Abhishek Khedkar, Lana Mineh, Ashley Montanaro, Katherine Inzani, Jonathan D. Hirst

**Affiliations:** † School of Chemistry, 6123 University of Nottingham, Nottingham NG7 2RD, United Kingdom; ‡ Physical & Theoretical Chemistry Laboratory, Department of Chemistry, 6396 University of Oxford, Oxford OX1 3QZ, United Kingdom; ¶ QuEra Computing Inc., Boston, Massachusetts 02135, United States; § Phasecraft Ltd., London W1T 4PW, United Kingdom

## Abstract

Targeted covalent inhibitors represent a promising class of drugs that form specific chemical bonds with their biological targets. There are a multitude of molecular systems where covalent inhibitors could address human health challenges. A high-level quantum chemical description of the formation of the critical covalent bond could provide new mechanistic detail and insights into how the mechanism is influenced by the surrounding biomolecular environment. However, accurately simulating the reactivity and binding specificity of such inhibitors remains a significant challenge. By leveraging advances in quantum computing hardware and algorithms, we discuss how quantum computing could benefit the design of targeted covalent inhibitors and enable more accurate simulations of protein–ligand interactions and accelerate *de novo* drug discovery.

## Graphical abstract

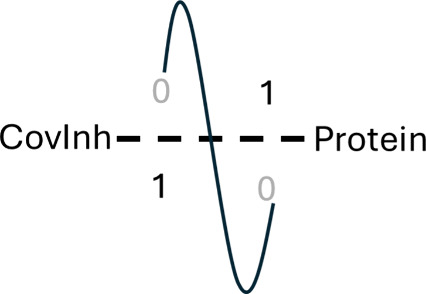

There is a resurgence of interest in targeted covalent inhibitors in drug discovery.^1^ The importance of this class of compounds is evident from the range of data in the community resource CovalentInDB 2.0,^2^ which features over 8300 experimentally confirmed covalent inhibitors, more than 110 distinct warhead chemistries, 386 protein targets amenable to covalent inhibition, and 75 targeted covalent inhibitors marketed as drug therapies. Recently approved covalent kinase inhibitors include ibrutinib for B-cell cancers^3^ and afatinib and osimertinib, which are treatments for non-small-cell lung carcinoma.^4,5^ One example which has been the subject of ongoing study is the covalent inhibition of cyclin-dependent kinase 12 (CDK12). This protein is being explored as a target for the treatment of myotonic dystrophy type I.^6^ One of the cysteine residues of CDK12 acts as a nucleophile to form a covalent bond with inhibitor molecules, as shown in Figure 1.

**Figure 1:**
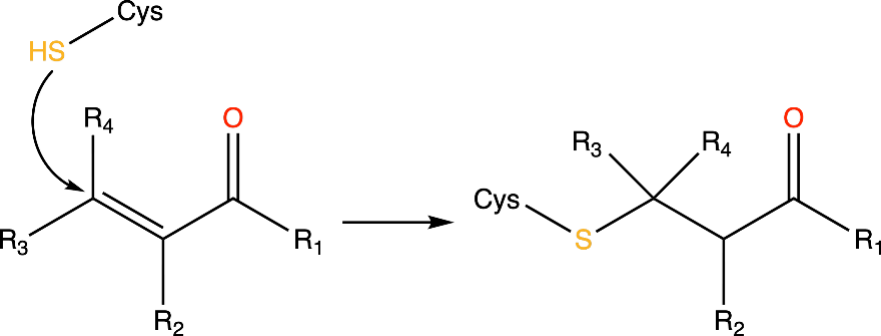
General reaction scheme of a thio-Michael addition, with the thiol (SH) group in cysteine (Cys) acting as a nucleophile.

Covalent inhibitors utilize a functional group with an electrophilic center to attack nucleophilic residues within a specific disease-relevant target protein.^1^ In particular, the formation of a covalent bond with its target means that strong potencies can be achieved with minimal effects experienced in the presence of competing cellular substrates, as well as stronger binding affinities beyond the noncovalent interactions involved in drug binding.^7,8^ These factors allow such covalent inhibitors to be administered in smaller and fewer doses, which can significantly improve patient compliance, limit the occurrence of unpredictable toxicities, and in many cases reduce both production and treatment costs.^1^

Covalent drug design, however, presents a challenging frontier for classical computational resources, as only the most advanced and poorly scaling quantum mechanical methods can fully capture the key interactions at the binding sites.^9,10^ Accurate modeling of bond formation and bond breaking demands a high-level quantum chemical treatment. The size of the simulation region required to study the binding between covalent inhibitor ligands and proteins severely limits the applicability of these methods, and approximations often have to be made which can suffer from spurious errors and numerical instabilities.^11^ This bottleneck in classical computations therefore hampers progress in the burgeoning field of covalent drug design.^12^

In the early 1980s, Feynman^13^ proposed that physics could be simulated using a universal quantum computer, a device that exploited inherently quantum phenomena such as entanglement. Rather than classical bits, which can only take values of either 0 or 1 and are the bedrock of conventional computers, quantum computers utilize qubits, which can exhibit superpositions of quantum states and therefore store an exponentially larger amount of information compared to classical bits. In principle, quantum computers could solve certain types of computational problems exponentially faster, and this promise is, in part, responsible for the rapid growth of the field of quantum computing at the start of this century. For example, quantum algorithms such as Shor’s algorithm for prime factorization^14^ and Grover’s algorithm for unstructured searches^15^ illustrate the potential speed-up offered by quantum computers for tasks that are computationally impractical with classical computers. Recently, quantum computers have been employed to simulate a range of chemical reactions, including the isomerization mechanism of diazene^16^ and the Diels–Alder reaction.^17^ Current research seeks to refine the formulation of chemical problems that may see early benefits, and advances in quantum computing hardware, algorithm development and software are pushing toward a practical advantage over conventional quantum computing simulations in the near term. One of the most fruitful areas for the promise of quantum computing to be realized is quantum chemical calculations.^18^

In this Perspective, we survey the main challenges commonly encountered in computational simulations of binding mechanisms of covalent inhibitors. To access the thermodynamics of binding using computational chemistry approaches, one needs both an adequate sampling of the relevant conformational space as well as a level of electronic structure theory that has a consistent accuracy across the reaction coordinate(s) of interest.^19^

## Simulation Challenges

The recent emergence of targeted covalent inhibitors in drug discovery presents new opportunities and challenges to the quantum chemical calculations of reactivity that support discovery efforts.^20^ Covalent bond formation between an inhibitor and its target enzyme occurs in a two-step process:

1 $$P+I\overset{k_{i}}{\underset{k_{-i}}{⇌}}\mathrm{P:I}\overset{k_{inact}}{\to }\mathrm{PI}$$

The inhibitor, I, binds to the target protein, P, forming a protein–inhibitor complex, P:I. The binding equilibrium constant, *K*_I_, defines the binding potency, with *k*_i_ (*k*_–i_) being the on (off) rate constant for the noncovalent binding between I and P. The rate of the second step, i.e., bond formation to form the covalently bound protein–inhibitor complex, PI, is denoted *k*_inact_. The efficiency of covalent bond formation is described by the ratio *k*_inact_/*K*_I_, which is a second-order rate constant, where *k*_inact_ is determined by the free energy of activation, $\Delta G_{inact}^{‡}$ .^21^

From the Eyring equation^22^

2 $$k_{inact}\propto \exp\left(\frac{-\Delta G_{inact}^{‡}}{RT}\right)$$

where *R* is the universal gas constant and *T* is the absolute temperature at which the reaction takes place. An error of just 5 kcal mol^–1^ in the calculation of $\Delta G_{inact}^{‡}$ would result in an error of 3 orders of magnitude in *k*_inact_ at room temperature, which could correspond to the difference between a selective and a nonselective inhibitor. Thus, as is widely recognized by the phrase “chemical accuracy”, an accuracy of 1 kcal mol^–1^ or better is needed for computational methods to be quantitatively useful in simulating the binding of targeted covalent inhibitors.

### State-of-the-Art Methods for Simulating Covalent Inhibitors

At the core of targeted covalent inhibitor design lies the need for a deep understanding of molecular electronic structure, which governs reactivity, binding, and selectivity. Hybrid approaches such as quantum mechanics/molecular mechanics (QM/MM) simulations have been instrumental in studying biomolecular interactions,^23,24^ and have benefitted from algorithmic improvements and classical computing hardware advances.^25^ Yet, achieving high accuracy remains computationally demanding, often requiring approximations that limit predictive power. In the current panoply of computational methods, QM/MM calculations are expected to provide the most accurate descriptions of covalent ligand binding that can be tractably computed. Mihalovits et al.^12^ cite 33 QM/MM studies of covalent inhibitors on a range of systems, including the inhibition of human cyclooxygenase-1 by aspirin^26^ and the inhibition of the SARS-CoV-2 main protease by a peptidyl Michael acceptor.^27^ QM/MM studies can provide insight into the nuanced interplay of entropic effects, conformational flexibility, covalent bond formation, and noncovalent interactions that govern the mechanisms of targeted covalent inhibition, for example, the binding of Bruton’s tyrosine kinase by a cyanoacrylamide derivative.^28^ There are many molecular systems where covalent inhibitors could address human health challenges and where a high-level quantum chemical description of the critical covalent bond could provide new mechanistic detail and insights into how the mechanism is influenced by the surrounding biomolecular environment.

To perform such QM/MM calculations, an optimal combination of the QM method and QM region is required. There can be a strong dependence between what is included within the QM region and the obtained energies.^29^ Larger QM regions tend to improve the accuracy of the QM/MM calculation but increase the overall computational cost. The choice of QM method can also have a dramatic effect on the simulation, with some QM methods being better suited for certain systems than others.^30^ As most QM/MM methods use statistical mechanics to obtain properties such as the free energy of the system,^31^ the amount of sampling also has an impact. Longer simulations provide greater sampling of the configurational space, increasing statistical convergence on the true free energy of the system. In QM/MM calculations, a trade-off between QM size, QM method, and simulation length has to be made. Often, a QM benchmark is performed to obtain the optimal QM method for the simulation, but accurate energies for the benchmark are difficult to obtain (*vide infra*). Ideally, this QM benchmark would not have to be performed, but there are currently no universal QM methods which are both cheap enough for use within QM/MM methods and accurate enough to capture the properties of the system correctly.

### Density-Functional Approximations

QM/MM calculations with density functional theory (DFT)^32,33^ are based on the assumption that the density functional approximations (DFAs) typically employed are sufficiently accurate for the description of the QM regions. Unfortunately, it is difficult to make such a general assessment, especially for “off-the-shelf” usages of DFAs, due to the nonsystematic nature in the construction of most commonly available DFAs. In a 2024 perspective, Santagati et al.^34^ assert that “current classical quantum chemistry algorithms fail to describe quantum systems accurately and efficiently enough to be of practical use for drug design”. While the previous statement might be a little too pessimistic, QM/MM simulations are often preceded by an extensive calibration exercise as quantitative accuracy can be elusive, even when using modern DFAs that are high up on Jacob’s ladder,^35^ due to the chemical complexity of the mechanisms.

Assessment of DFAs over large data sets of structural and energetic properties is well-established as an approach to test the strengths and weaknesses in DFAs. For example, the GMTKN55 benchmark database has been used to assess 217 variations of dispersion-corrected and -uncorrected DFAs.^36^ A good correlation between simulations and experiments for a particular system may well be due to a fortuitous cancellation of errors. For example, many DFAs struggle to describe bond dissociation and how electrons localize on ions as the interatomic distance increases,^37^ while which DFA gives the best treatment of CH−π interactions is still a topic of current discussion.^38,39^ Typically, to describe these complex processes quantitatively requires either the use of hybrid DFAs that mitigate the self-interaction error found with standard GGAs and meta-GGAs, which are very computationally demanding, and/or an accurate description of long-range interactions beyond interatomic or electron density-based pairwise approximations.^40^ Despite the development of many-body dispersion correction schemes^41,42^ and van der Waals DFAs,^43^ accurately capturing both covalent and noncovalent interactions in complex biomolecular systems, such as targeted covalent inhibitors, remains a significant challenge for DFT methods.

### Correlated Wave Function Methods

Accurate and systematically improvable QM/MM calculations of reaction barriers in biomolecular systems require correlated *ab initio* methods.^44–46^ For instance, a projector-based embedding of a wave function method in DFT gave a spread in barrier heights of just 0.3 kcal mol^–1^ compared to a 13 kcal mol^–1^ spread calculated at the DFT/MM level, suggesting that DFA dependence can be eliminated by incorporating higher-level methods.^46^ Sophisticated wave function methods such as coupled cluster (CC),^47^ and complete active space self-consistent field (CASSCF) with complete active space second-order perturbation theory (CASPT2)^48,49^ can be employed for systematic improvements. Multiconfigurational methods, such as CASSCF, can describe bond breaking and formation that are essential to study covalently bonded inhibitors and electronically excited states. The component of dynamical electron correlation absent in a CASSCF wave function can be accounted for by multireference methods, such as in the CASPT2 approach.

Bistoni et al.^47^ employed domain-based local pair natural orbital CC energies evaluated on DFT/MM single-point structures to study two enzyme-catalyzed reactions. They found that energy barriers, computed using different DFAs, gave qualitatively different results (variations of the order of 10 kcal mol^–1^ for both reactions), and concluded that more reliable QM/MM predictions are obtained when CC is employed as the QM component. QM/MM calculations with multiconfigurational wave function approaches employed as the QM method have also been applied to study photoreactions in solvated molecules and in proteins. For example, the photoinduced ring-closure/opening and isomerization reactions of a photochromic indolylfulgide in acetonitrile solution were studied by Wang et al.^48^ at the CASSCF/MM level of theory, with multistate CASPT2 used to re-evaluate CASSCF energies. Pan et al.^49^ employed CASSCF/MM calculations, with extended dynamically weighted CASPT2 used to re-evaluate CASSCF energies, to study excited-state proton transfer and the photoisomerization processes of the red fluorescent protein mKeima. However, such robust CC- or CAS-based QM/MM calculations are not fast enough or sufficiently automated to be applied routinely for screening compounds in a lead optimization study to targeted covalent inhibition of biomolecules.

### Fitted Density-Functional Approximations

DFAs can be improved by adjusting an approximate form by fitting to experimental data or higher-level quantum calculations, with exact constraints incorporated to guide this parametrization. This process can be both data-driven and manually adjusted based on a physical and chemical understanding of the system. Machine learning can also be used to optimize DFA fitting beyond human-guided attempts. Modern machine-learned DFAs offer promise for future enhancements;^50,51^ the DM21 DFA showed general success for the data set on which it was trained.^50^ However, such foundational models require augmenting with a broader set of data for a specialized task or to be applicable outside of their training domain. Such limitations have been highlighted for DFAs such as DM21, which was shown to not extrapolate for transition metals.^52^ To this end, full configuration interaction and other (near-)­exact methods can, of course, be used to fit DFAs.

For generating the requisite data set, advanced computational techniques will be required beyond current capabilities, in either conventional or quantum computing. Within conventional computing, as well as a move into exascale computing, algorithmic advancements continue; for instance, a presentation at the *QM in Pharma* meeting in London in September 2024 ^53^ boasted a speed-up of over 1000 times for a conventional DFT calculation on a large molecule using ORCA 6 (released in 2024) compared to ORCA 2.4 (released in 2004). Meanwhile, the quantum computing community is preparing for a transition to fault-tolerant computations, developing both algorithms and hardware that can move beyond current noisy intermediate-scale quantum (NISQ) techniques. While the limitations of the NISQ era regarding system size and fidelity severely restrict practical application of quantum computing to quantum chemistry currently, the availability of fault-tolerant quantum computers and algorithms will enable (near-)­exact quantum chemical calculations.

## Recent Quantum Computing Advances and Outlook

In its early stage, quantum computing was deemed as a theoretical construct whose physical realization was precluded by taxing qubit-fidelity requirements for useful applications, well beyond those attainable in the laboratory. This vision was challenged in 1995 when Shor^54^ theoretically showed that physical qubits featuring error rates below a threshold can encode a computational space with an exponentially suppressed error rate. The advent of physical platforms capable of hosting qubits with error rates approaching the error threshold for quantum error correction has revived the interest in the pursuit of scalable fault-tolerant quantum computers and in seeking useful applications in near-term NISQ devices.^55^ *In lieu* of exhaustively reviewing current quantum computing platforms, we highlight the ones which have shown significant progress in recent years.

### Solid-State Platforms

Solid-state quantum circuits rely on nanostructures connected to each other in a hardwired fashion akin to classical electronic integrated circuits. The advantages of this type of architectures include fast gates, possibility of industrial fabrication, and availability of control equipment.^56^ However, solid-state devices suffer from important shortcomings: limited connectivity due to their hardwired nature, and potential loss of scalability as all qubits and their junctions must be individually controlled. The leading solid-state platform is superconducting qubits, among which transmons constitute the current leading architecture^57–59^ in terms of gate fidelity. Semiconductor quantum-dot architectures have recently gained some traction due to their prospective scalability advantages over transmon qubits but the technology maturity is relatively low.^60,61^

### Atoms, Ions, And Molecule-Based Platforms

The technological challenge of fabricating identical qubits in solid-state platforms is bypassed in these architectures by employing atoms, ions or molecules instead, at the expense of engineering control and interaction protocols between these units. In real space, qubits are addressed by means of tunable electromagnetic fields (traps) and mechanical fluctuations of physical qubits are minimized by laser cooling. The leading platforms within this family are cooled trapped ions and neutral atoms.^62–64^ In the former, an ordered array of ions is trapped and its mutual repulsion leads to the emergence of mechanical modes, which enable full connectivity of quantum units^64^ but also gives rise to scalability issues. The situation with neutral atom platforms is opposite; Rydberg atoms are trapped in optical fields, enabling better scalability. However, their interactions are relatively short-ranged, which limits the quality of pairwise operations. Trapped-ion systems have demonstrated unparalleled qubit fidelity and all-to-all connectivity in small modules, but their slower gate speeds and multitrap complexity currently limit practical scaling. Neutral-atom quantum computing has rapidly advanced from academic prototypes to commercially accessible platforms, demonstrating hundreds to thousands of qubits with promising trajectories toward scalable, fault-tolerant architectures.

### Optical Platforms

In this category, quantum information is encoded in light waves propagating in certain optical modes.^65^ One of the main characteristics of light that would make it amenable to a quantum computing scheme is its long coherence time. However, the difficulty in engineering nonlinear interactions between optical modes^66^ necessary for the implementation of entangling gates, as well as the short time scales at which these platforms operate, render an error-corrected and gate-based model of computation challenging.^55^ Photonic systems excel at long-distance entanglement and quantum networking but remain at an exploratory stage for general-purpose quantum computation, with significant resource overhead required for reliable multiqubit operations.

The maturity landscape of quantum hardware also varies widely: superconducting circuits lead in commercial availability, trapped ions set the benchmark for fidelity, photonics pioneer networking applications, and neutral atoms are emerging as the most scalable contenders for future fault-tolerant systems, as compared in Table 1. A more detailed comparison between existent quantum computing platforms can be found in The Quantum Insider.^67^

**Table 1:** Comparison of Major Quantum Computing Platforms by Maturity, Strengths, and Challenges

Platform	Maturity Level	Strengths	Challenges
Superconducting	Most mature, available with more than 100 qubits	Fast gates, industrial backing	Cryogenic, wiring bottlenecks
Trapped ions	Commercially available, 20–50 ions	High fidelity, all-to-all connectivity	Slow gates, modular scaling
Photonic	Early stage, exploratory	Long-distance transmission, natural networking	Probabilistic gates, high loss
Neutral atoms	Rapidly scaling and maturing; available for analog and digital modes with >200 qubits	Large arrays (demonstrations with >1000 qubits) flexible connectivity, long coherence	Laser complexity, gate speed

### Seeking Quantum Utility

There is a myriad of classically hard problems which have been suggested to benefit from quantum computers, including cryptography, chemistry and materials science, optimization, machine learning, database searching, and protein folding.^68^ However, considering the overhead associated with classical data loading and the slower gate execution afforded by quantum computers compared to their classical counterparts, the most promising applications for these devices should consist of small data and computationally intensive problems,^68^ for which a significant quantum speed-up can compensate for fault-tolerance overheads. Specific instances where this is the case are quantum problems in chemistry and material science. In particular, covalent inhibitor modeling requires a QM framework to address the inaccurate description through traditional MM, which fails to describe covalent interactions and reaction mechanisms that involve bond breaking and finding transition state structures.^9^

Applying standard algorithms directly to full-scale molecular simulation tasks will necessitate the use of fault-tolerance. However, when full error-correction overhead is included in cost estimation, it is evident that simulation of concrete molecular instances are beyond today’s quantum hardware capabilities.^69–72^ Due to the entwined nature of hardware and algorithm development it cannot be said one or the other is the dominant bottleneck. For example, the original motivation from quantum computing came from algorithms developed in the 1980s, devices are now being constructed, some tasks can be run on a quantum device and algorithmic advances can bring down resource requirements significantly (many orders of magnitude - with further gains likely), yet improved hardware is still needed. The convergence of hardware design and algorithm design targeted to key requirements of an end-use application is the main goal, where any aspect can become a potential bottleneck. If wanting to use near-term hardware, innovative algorithmic methods should be developed and deployed to achieve useful molecular computations on such devices.

### Algorithmic Advancements

The most widely studied algorithm on near-term quantum hardware for predicting ground-state properties is the variational quantum eigensolver (VQE),^73,74^ which is based on the Rayleigh–Ritz variational characterization of the Hamiltonian (Ĥ) eigenvalues, as shown in eq 3:

3 $$\lambda_{0}\left(Ĥ\right)=\underset{\left|\psi \right\rangle }{\min}\left\langle \psi \right|Ĥ\left|\psi \right\rangle$$

Unlike in classical variational algorithms, in VQE the state |ψ⟩ is generated, and its energy E(|ψ⟩)=⟨ψ|Ĥ|ψ⟩ , with respect to Ĥ , is measured on a quantum computer. This requires a suitable, efficiently preparable class of quantum states, |ψ­(θ)⟩, to be chosen with parameter vectors θ over which to optimize.

Various *ansätze* have been proposed for VQE algorithms in order to improve the accuracy of the quantumly computed trial state, including hardware-efficient,^75^ unitary coupled cluster,^76^ Hamiltonian variational,^77^ Jastrow,^78^ and adaptive derivative-assembled pseudo-Trotter (ADAPT)^79^ *ansätze*. The Hamiltonian variational *ansatz* (HVA) is an example of VQE which constructs the trial wave function directly based on the terms in Ĥ . HVA allows for a more physically motivated representation of |ψ⟩, often requiring fewer parameters than more generic *ansätze*, such as the unitary CC or hardware-efficient *ansätze*. Furthermore, HVA naturally incorporates symmetries of the system, which can result in potentially faster convergence of calculations. HVA could, therefore, allow more accurate modeling of electronic interactions and bond formation processes, which are crucial for understanding the reactivity and specificity of covalent inhibitors with their targets.

As well as by making improvements to the quantum algorithm itself, significant reductions can be achieved in complexity by considering the overall problem of modeling materials or chemical systems holistically. Developing an integrated framework which considers the choice of active space, Fermionic encoding, and algorithm design together can enable substantial savings compared with an approach in which each is considered separately. By following this philosophy, a circuit depth improvement by up to 6 orders of magnitude compared with the best previous quantum algorithm resource estimates has been achieved for implementing a single layer of VQE for the transition metal oxide strontium vanadate.^80^ A wider perspective on recent developments has been provided by Daley et al.^81^

In addition, interesting ideas have recently emerged in which the quantum processor is regarded as a classical pipeline enhancer, rather than the sole device executing the computation,^37,82,83^ which constitute a prospective avenue to bridge the gap to useful applications before error-correction capabilities are incorporated in quantum computing platforms. We discuss below one approach of this form which we are pursuing.

### Applications for Targeted Covalent Inhibitors

The focus of QM/MM studies of covalent inhibitors has mainly been on elucidating reaction mechanisms. However, there are wider applications. The versatility of QM/MM approaches extends to investigation of the effects of warhead modulation on inhibition rate, the development of QM/MM-based scoring functions, and the calculation of affinity and selectivity through the use of thermodynamic integration calculations. We discuss some of these in the context of recent studies using conventional computers. We then survey recent quantum computing work in this area and suggest some likely future developments.

Rocelitinib-like covalent inhibitors of the epidermal growth factor receptor have been studied using QM/MM,^84^ addressing questions including the influence of the location of the electrophile in the ATP binding site and the role of the acrylamide conformation, which can be either s-*cis* or s-*trans*. Empirical force field molecular dynamics simulations were performed to sample reactive conformations for subsequent QM/MM calculations. The QM region was treated using the M06–2X hybrid meta-GGA and comprised over 200 atoms, with the majority described with the 6–31G­(d) split-valence Pople basis set and the rest with a smaller basis. Two different possible mechanisms of the inhibition of the epidermal growth factor receptor by afatinib have been investigated using QM/MM,^24^ where the QM treatment was based on the Pairwise Distance Directed Gaussian modification of the semiempirical PM3 method.

Free-energy perturbation/umbrella sampling calculations have been used to simulate the covalent inhibition of the SARS-CoV-2 main protease, also known as the 3-chymotrypsin-like protease, by the nitrile electrophile group and by the less reactive alkyne group.^85^ An empirical valence bond approach, calibrated by *ab initio* calculations was then applied. The time-dependent half-maximal inhibitory concentration (IC_50_), which can be measured experimentally, was studied using a kinetic simulation approach. Related systems have been studied^86^ using alchemical techniques (imposing artificial modifications) with an empirical MM force field and the thermodynamic integration protocol.

Some work on the application of quantum computing to covalent inhibitor drug discovery has focused on the resources required for fault tolerant computations, i.e., the number of qubits and the number of gates. Blunt et al.^9^ considered the binding of the anticancer drug ibrutinib to Bruton’s tyrosine kinase. A region of over 100 atoms was defined for treatment at a QM level. An important strategy is the definition of an active space, whereby a subset of ‘active’ electrons and a subset of ‘active’ orbitals are identified. All possible configurations, i.e., arrangements of the electrons in the orbitals, are generated within the active space. Thus, one is performing full configuration interaction within the active space, with the associated exponential scaling. An active space of 20 electrons in 20 orbitals represents the limit of tractability on current conventional computers, without resorting to bespoke supercomputers or classically approximate algorithms.^87,88^ While there are various techniques for tackling larger active spaces, the desire for a fully general approach motivates the application of quantum computing. For an active space of 32 electrons and 32 orbitals, Blunt et al.^9^ suggest some algorithmic enhancements, including Hamiltonian truncation and sparse qubitization, to the quantum phase estimation approach to reduce the resources to 10^3^ logical qubits, and 10^10^ *T* gates. Enabled by the qubitization method, the Hamiltonian’s energies are found by performing quantum phase estimation on a so-called walk operator, which can be implemented with many fewer *T* gates than solving the Hamiltonian simulation with a standard Trotterized time evolution. Furthermore, the sparsity of the Hamiltonian matrix is exploited by discarding terms with two-body coefficients below a chosen threshold.

Recognizing limited qubit availability and measurement noise on current quantum hardware, much more modest resources are used in computational workflows employing an actual quantum computer. Sotorasib is a covalent inhibitor of the Gly12Cys mutant of the Kirsten rat sarcoma viral oncogene, an important cancer target. Li et al.^89^ have studied this system using a hybrid quantum computing workflow, with VQE, which has implemented molecular forces for a QM/MM calculation. This involves measuring the one- and two-body reduced density matrices of the active space, which was chosen to be 2 electrons and 2 orbitals, to render the calculation tractable on a 2-qubit superconducting quantum device. A hardware-efficient *R*_*y*_ *ansatz* with a single layer was employed as the parametrized quantum circuit for VQE, with a standard readout error mitigation to enhance the accuracy of the measurement results.

## Outlook

Quantum chemistry is a natural fit to the quantum computing paradigm, with the benefit being most obvious for highly correlated systems. In a bond formation process involving a conjugated Michael acceptor, geometric distortions may well lead to a variation in correlation energy along the reaction pathway. Thus, for modeling covalent inhibition, there is a clear need for a quantum chemical approach that is as accurate as possible. Quantum computing is still a nascent technology which has not yet been used for practical applications,^90^ but the speed of development of both hardware and algorithms is cause for optimism.

In this Perspective, we have highlighted some recent algorithmic developments, and this continues to be a very active area of research.^91,92^ We are working on developing and applying a new hybrid quantum-classical approach to the many-body electron structure problem, namely quantum-enhanced density functional theory (QE-DFT).^37^ QE-DFT combines the complementary strengths of DFT and quantum computation, and rather than using a quantum computer to find the ground state itself, it is instead used to approximate the exchange-correlation functional.^37^ The quantum-enhanced DFA is then fed into a classical DFT iteration; the quantum computer is thus not tasked with finding the ground state itself, but rather with steering the DFT iteration toward an accurate solution. QE-DFT usually achieves higher accuracy over both standalone Hartree–Fock or purely quantum approaches such as quantum VQE.^37^ Moreover, QE-DFT does not necessarily rely on quantitatively accurate quantum computations so could provide a path (Figure 2) to quantum advantage even when the quantum hardware is noisy.

**Figure 2:**
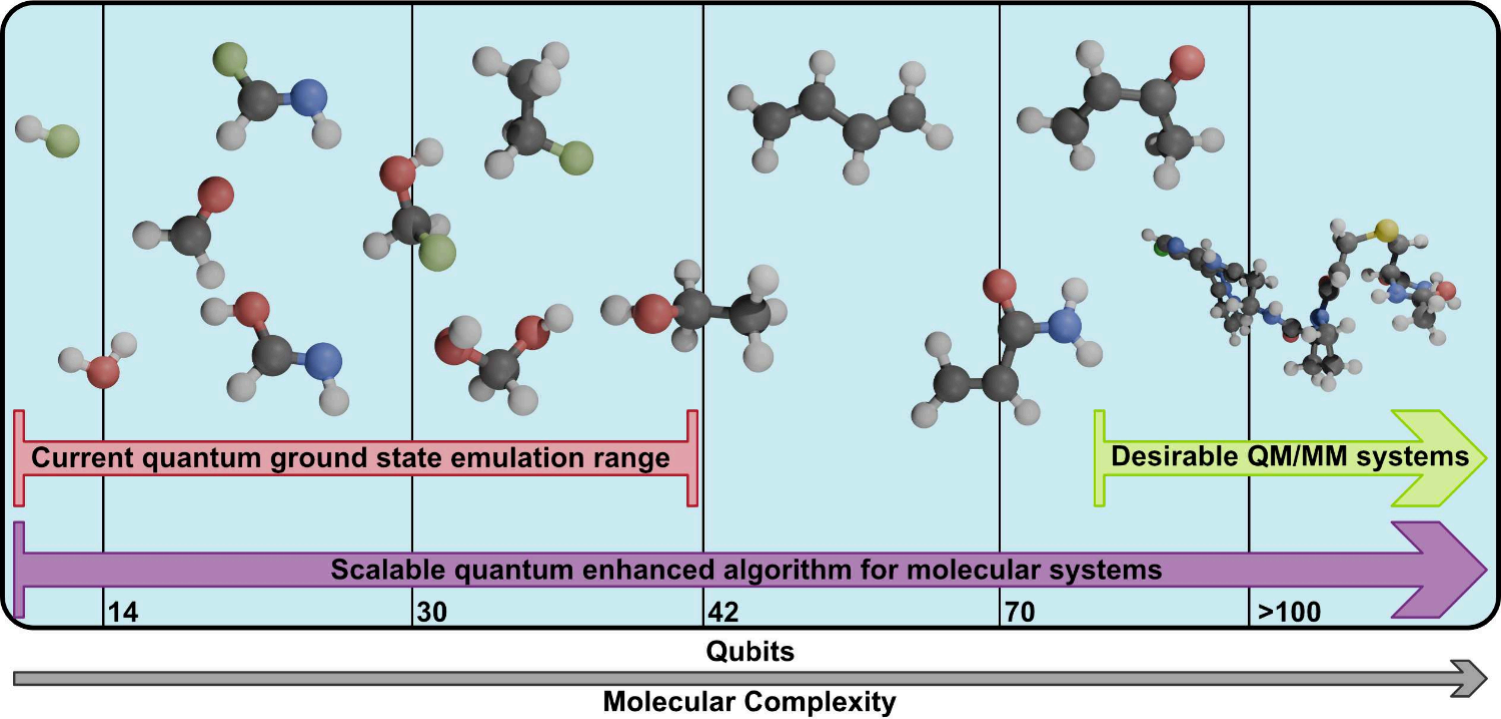
Roadmap toward the quantum simulation of a covalent inhibitor, from small model systems such as lithium hydride, on the far left-hand side, through to acrylamide at around 70 qubits, and onto a full covalent inhibitor ligand bound to a cysteine amino acid on the far right-hand side. Emulation on current classical hardware is feasible for up to 40 qubits.

Systematic discovery of targeted covalent inhibitors calls for *ab initio* simulations that simultaneously deliver quantum-level accuracy for covalent transition states, accommodate pharmacologically relevant system sizes, and span physiologically meaningful reaction time scales. Conventional meta-GGAs and hybrid DFAs can systematically mis-predict activation barriers, hampering routine conformational scans. CC with with single, double, and perturbative triple excitations [CCSD­(T)] can potentially achieve the desired accuracy, but its $O\left(N^{7}\right)$ scaling is prohibitive for screening campaigns. Multireference methods such as CASSCF capture components of correlation yet suffer from an exponential growth of configuration space. QE-DFT offers a promising alternative: its functionals are trained on data sets obtained from quantum-hardware simulations and implicitly retain functional forms that compare to routine DFT computations. In principle, QE-DFT makes the tractable exploration of larger and more electronically complex chemical spaces feasible, accelerating the search for next-generation targeted covalent inhibitors.

Today, for a given biomolecular system of interest, one would reach for conventional computing and, depending on the resources available, a variety of contemporary computational chemistry methods. However, in the context of the development and demonstration of the tools for the future, there is a real prospect that one will be able to slot a quantum-enhanced DFA, generated from data from quantum computations, directly into well-established QM/MM workflows.

## Data Availability

No new data are associated with this Perspective.
